# Insomnia and job stressors among healthcare workers who served COVID-19 patients in Bangladesh

**DOI:** 10.1186/s12913-023-09464-x

**Published:** 2023-05-23

**Authors:** Farzana Rahman, Koustuv Dalal, Mehedi Hasan, Tariful Islam, Samiha Nahar Tuli, Asma Akter, K M Tanvir, Khairul Islam, Ashikur Rahman, Mohammad Hayatun Nabi, Mohammad Lutfor Rahman, Mohammad Delwer Hossain Hawlader

**Affiliations:** 1 Department of Public Health, North South University, Dhaka, 1229 Bangladesh; 2 Armed Forces Medical College, Dhaka, 1206 Bangladesh; 3 School of Health Sciences, Division of Public Health Science, Mid Sweden University, Sundsvall, Sweden; 4 Institute of Statistical Research and Training (ISRT), University of Dhaka, Dhaka, 1000 Bangladesh

**Keywords:** Insomnia, COVID-19, Healthcare worker, Job Stressor, Bangladesh

## Abstract

**Background:**

The global outbreak of COVID-19 has created unprecedented havoc among health care workers, resulting in significant psychological strains like insomnia. This study aimed to analyze insomnia prevalence and job stressors among Bangladeshi health care workers in COVID-19 units.

**Methodology:**

We conducted this cross-sectional study to assess insomnia severity from January to March 2021 among 454 health care workers working in multiple hospitals in Dhaka city with active COVID-dedicated units. We selected 25 hospitals conveniently. We used a structured questionnaire for face-to-face interviews containing sociodemographic variables and job stressors. The severity of insomnia was measured by the Insomnia Severity Scale (ISS). The scale has seven items to evaluate the rate of insomnia, which was categorized as the absence of Insomnia (0–7); sub-threshold Insomnia (8–14); moderate clinical Insomnia (15–21); and severe clinical Insomnia (22–28). To identify clinical insomnia, a cut-off value of 15 was decided primarily. A cut-off score of 15 was initially proposed for identifying clinical insomnia. We performed a chi-square test and adjusted logistic regression to explore the association of different independent variables with clinically significant insomnia using the software SPSS version 25.0.

**Results:**

61.5% of our study participants were females. 44.9% were doctors, 33.9% were nurses, and 21.1% were other health care workers. Insomnia was more dominant among doctors and nurses (16.2% and 13.6%, respectively) than others (4.2%). We found clinically significant insomnia was associated with several job stressors (p < 0.05). In binary logistic regression, having sick leave (OR = 0.248, 95% CI = 0.116, 0.532) and being entitled to risk allowance (OR = 0.367, 95% CI = 0.124.1.081) showed lower odds of developing Insomnia. Previously diagnosed with COVID-19-positive health care workers had an OR of 2.596 (95% CI = 1.248, 5.399), pointing at negative experiences influencing insomnia. In addition, we observed that any training on risk and hazard increased the chances of suffering from Insomnia (OR = 1.923, 95% CI = 0.934, 3.958).

**Conclusion:**

It is evident from the findings that the volatile existence and ambiguity of COVID-19 have induced significant adverse psychological effects and subsequently directed our HCWs toward disturbed sleep and insomnia. The study recommends the imperativeness to formulate and implement collaborative interventions to help HCWs cope with this crisis and mitigate the mental stresses they experience during the pandemic.

## Introduction

The China office of the World Health Organization (WHO) noticed some atypical pneumonia-like cases in Wuhan city of Hubei province on 31st December 2019 [1], which soon turned into an epidemic. Despite numerous efforts to limit its transmission, this deadly virus propagated across the planet. In March 2020, the outbreak was announced as a pandemic due to its devouring extent in more than 110 countries [2]. The thriving number of contagions and mortality brought an overwhelming burden on the health system and health care professionals globally. In Bangladesh, the Institute of Epidemiology, Disease Control and Research (IEDCR) identified the first three cases of COVID-19 on 8th March 2020 [3]. According to the WHO-Bangladesh COVID-19 situation report (Mortality and Morbidity Weekly Update – 44), there were 5,09,148 RT-PCR confirmed positive cases in Bangladesh as of 27 December 2020, causing 7,452 deaths with a case fatality rate of 1.46% which accounted for 0.64% of the global disease burden [4]. Being a low- and middle-income country in Bangladesh, healthcare workers (HCW) are experiencing immeasurable workloads and psychological burdens to mitigate the ruinous spread of this disease due to insufficient workforce and their disproportionate distribution, which ultimately results in numerous adverse outcomes [3, 5].

HCWs are the most endangered part of the population to develop psychological distress and stress-related symptoms like insomnia. Previous studies on reactions to SARS, 2003 revealed outrageous psychological impacts on HCWs, interacting closely with infected patients and reported a higher incidence of insomnia, stress, and depression, leading them towards fatigability in work and sometimes intending to resign from jobs [6]. As frontline caregivers, they have to expose themselves directly or indirectly to the highly contagious virus while handling severely ill patients and managing extremely traumatized people in crowded settings [7]. While defending against this havoc, many negative emotions and mental health issues, including anxiety, depression, panic attacks, and sleep disturbance, are cultivating amid the HCWs. However, the most exhausting among these effects is disrupted circadian rhythm and impaired sleep, when unimpeded sleep is crucial to acclimatize themselves to the pandemic and obscurity about the future [8, 9].

Lin and his colleagues investigated the impact of the COVID pandemic on the sleep pattern of Chinese HCWs [10]. The investigation revealed a higher rate of clinically significant insomnia (20%) over acute stress and anxiety. Compared to the pre-pandemic period, the investigators reported a 37% increased rate of clinical insomnia during the COVID-19 pandemic [10]. In another study in Turkey, 50.4% of the HCWs were experiencing insomnia, with predominance among female workers [11].

Bangladesh is one of the most densely populated countries in the South Asian region with a lacking health care system, particularly the mental health sector, which is considered the missing brick of the health system. Moreover, the ambiguity of this pandemic, working in the isolation units with a scarcity of proper protective measures and witnessing the death of colleagues, has induced adverse psychological effects on HCWs, consequently driving them towards depression, anxiety, and sleep disturbance [12]. The constrained, overburdened work schedule of the frontline fighters exacerbated these disruptions. Sleep disorders, especially insomnia, usually persist long, even after abolishing any pandemic, which acts as precursors of chronic insomnia and other mental health disorders [10]. This study is noteworthy because it is the first to document the prevalence of insomnia and associated job stressors among frontline fighters of Bangladesh in response to the COVID pandemic and the first to examine the severity of insomnia based on the degree of threats of being infected with the coronavirus.

## Methodology

From January to March 2021, an analytical cross-sectional study was conducted in Dhaka city of Bangladesh, among Healthcare professionals working in COVID-19 isolation and treatment facilities. The sample size calculation was done considering 90% power. Initially, the calculated sample size was 454, counting the 44.2% prevalence obtained from a relevant study [13]. From around 50 COVID-19 dedicated hospitals, we selected 25 conveniently. Our inclusion criteria were all the health professionals (doctors, medical assistants, nurses, midwives, cleaners), irrespective of age and sex, working in the selected hospitals in Dhaka during the study period. We excluded the HCWs who were unwilling to participate in this study.

A structured questionnaire including three domains for socioeconomic variables, Insomnia Severity Index (ISI) scoring by A-5 point Likert scale, and job stressors has been developed and finalized after pretesting. The questionnaire was undertaken to check for the validity, appropriateness, and consistency of the variables used in the study. The questionnaires were initially prepared in English and then translated into Bengali. The rate of ISI was measured by the validated ISI questionnaire [14, 15]. The usual recall period is the “last month,“ and the dimensions evaluated are the severity of sleep onset, sleep maintenance, and early morning awakening problems, sleep dissatisfaction, interference of sleep difficulties with daytime functioning, noticeability of sleep problems by others, and distress caused by the sleep difficulties [13]. As the aim was to identify the prevalence and factors associated with insomnia, subjects scoring more than 15, i.e., evaluated with mild to severe insomnia, were not given any interventions.

Face-to-face interviews were conducted to collect data. The authors themselves conducted those interviews, and the hospitals were distributed among them according to their convenience. Informed written consent was taken from participants before the interview ensuring strict data confidentiality. According to the respondents ' preferences, the study used both the validated English and Bengali versions of the questionnaire. Throughout the data collection period, the consistency and competency of the collected data were checked regularly by the supervisors. The authors also started entry and cleaning procedures alongside data collection. Therefore, the chances of missing or irrelevant data were much less. Each questionnaire was rechecked to see whether appropriately filled or not, and only completed questionnaires were selected for the final analysis.

### Independent variable

Several independent variables were included in this analysis. Gender of HCWs, religion categorized as Islam and non-Islam (Hinduism, Buddhism and Christianism), the highest education level (graduate or undergraduate), designation of HCWs (doctors, nurses and others, which means any of the health care workers including SACMO, midwife, paramedics, cleaners). Marital status was sorted as married and others (never married, divorced or widow) and the participants’ smoking history was incorporated. Whether they were identified as COVID positive cases and whether suffering from any chronic disease (lasting longer than six months) or not, any training on COVID-19 was provided to the participants and was encountered as independent variables here.

To assess the association of insomnia with job stressors, several variables were evaluated concerning HCWs’ jobs after consulting with HCWs of different designations and further validated by face validation by experts and reliability was checked by Cronbach Alpha with a score of 0.836. Type of employment (permanent - not having a predetermined end date to employment and short-term contractual) employed for < 1 year, during the pandemic to serve the COVID-19- infected patients only), which showed the more profound concern about the continuity as well as the permanence of the current job, the greater the chance of insomnia among participants. Whether they were getting any pandemic-specific service benefits or not, e.g., added remuneration, health insurance, risk allowance for working in a highly contagious unit, provision of exceptional nutritious food to maintain their health and wellbeing, separate accommodation facility and arrangement of proper PPE to protect themselves from contagion and to restrain themselves from unintended transmission of contaminants to their kith and keen, are the variables assessed in this study. Job stressor predictors, e.g., changes in working hours (augmentation/reduction/ unaltered), availability of sick leave, and workload due to overtime duty to cope with the overwhelming burden of COVID patients, are also evaluated here. They were also asked about any training programs regarding the risks and hazards of COVID-19, which should enable them to perceive the harshness of this pandemic and safeguard themselves from this viral attack. These job stressors were measured by a 13 items questionnaire with dichotomous outcomes [16, 17]. The items were adapted from previously done studies related to almost all relevant components of the job life which cause stress in some way or another other such as type of employment, service benefit, overtime, changing working hours, risk allowance, PPE use and training [18].

### Dependent variables

Insomnia is a subjective disorder which is characterized by a poor or total lack of sleep. Insomnia initiates significant health hazards, including fatigue, lack of concentration, reluctance to work, and many more, mostly remaining neglected and untreated. A 5-point Likert scale with a total score ranging from 0 to 28, comprising seven items questionnaire, was used to assess the prevalence of insomnia among the study participants. The assigned scale was interpreted as the absence of Insomnia (0–7); sub-threshold Insomnia [8–14]; moderate clinical Insomnia [15–21]; and severe clinical Insomnia (22–28). A cut-off score of 15 was initially proposed for identifying significant insomnia [19].

### Statistical analyses

Univariate analyses to assess differences in demographic variables and job stressors among frontline health care workers were conducted using percentage distribution, and bivariate analysis using the chi-square test between insomnia (dependent variable) and all the variables of the other two domains was also done. Also, we conducted an adjusted logistic regression model for all the independent variables. We set the statistical significance (p-value) of < 0.05. Finally, we included statistically significant variables (5% significance level) from the adjusted logistic regression model. Also, adjusted odds ratios (AOR) and their 95% confidence intervals (CIs) were used as indicators for the strength of the association. All the statistical analyses were performed using SPSS software (version 25.0).

## Results

The final sample included 454 Bangladeshi healthcare practitioners (61.5% females). Concerning professions, 204 (44.9%) were doctors, 154 (33.9%) were nurses, and other health care workers were 96 (21.1%). More prevalent Insomnia was found among doctors (n = 33, 16.2%) and among the male HCWs (n = 25, 14.3%). The number of graduates and above participants was 310 (68.3%) with a higher prevalence of (n = 43, 13.9%), and undergraduate participants were 144 (31.7%). Compared to married participants (n = 28, 10.9%), those who never got married, divorced and widowed (n = 30, 15.8%) were more affected by COVID-19-induced insomnia. Most participants were followers of Islam (n = 342, 75.3%), but a higher manifestation of insomnia (n = 16, 14.3%) was found among non-Islamic believers. 361(79.5%) of the participants received training on COVID-19 and Insomnia was more prone among the trained professionals (n = 48, 13.3%). Among the 454 participants, 138 (30.4%) were diagnosed as COVID-19-positive previously and the symptoms of insomnia invaded n = 23 (16.7%) of those positive cases. Fortunately, 316 (69.6%) have never been diagnosed as positive. Among the HCWs, 77 (17%) have a chronic disease, whereas (377, 83.0%) have no such disease & appear less prone (n = 46, 12.2%) to develop Insomnia. As displayed in Table 1, among the 454 HCWs, (n = 67, 14.8%)) were smokers with clinically significant insomnia amid (n = 10, 14.9%).

**Table 1:** Association between insomnia and baseline characteristics

Questions on	Total Number of Observation N	Insomnia n (%)	p-value
*Gender of the participant*			
Male	175	25 (14.3)	0.445
Female	279	33 (11.8)	
*Designation of the participant*			
Doctor	204	33 (16.2)	0.014**
Nurse	154	21 (13.6)	
Others	96	4 (4.2)	
*Highest educational level*			
Undergraduate	144	15 (10.4)	0.305
Graduate and above	310	43 (13.9)	
*Marital Status*			
Married	257	28 (10.9)	0.170
Others	197	30 (15.8)	
*Religion of the participant*			
Islam	342	42 (12.3)	0.581
Non-Islam	112	16 (14.3)	
*Have you ever received training regarding COVID 19*			
Yes	361	48 (13.3)	0.512
No	93	10 (10.8)	
*Have you ever been diagnosed with COVID 19 disease*			
Yes	138	23 (16.7)	0.101
No	316	35 (11.1)	
*Do you have any chronic disease*			
Yes	77	12 (15.6)	0.418
No	337	46 (12.2)	
*Smoking history*			
Yes	67	10 (14.9)	0.568
No	387	48 (12.4)	

***= p < 0.001; **= p < 0.05

Table 2 shows the interrelationship between job stressors and Insomnia among HCWs during COVID-19. Frontline fighters (n = 248, 54.6%) who have permanent jobs and (n = 429, 94.5%) provided with written appointment letters were overwhelmed by the stress regarding their continued provision of health care service and were more prone to develop insomnia showed a prevalence of 14.1% and 13.1%, respectively. In addition, the provision of overtime night duty (n = 29, 13.6%) and not getting sick leave (n = 38, 25.3%) aggravated their work-related oppression and pushed them towards insomnia. Insomnia prevailed among those who did not get any accommodation (n = 34, 17.6%), exceptional nutritious food (n = 37, 14.3%) or any service benefit (n = 26, 21%) from the employer for working in a highly infectious unit, and risk allowance/insurance for a family if infected (n = 50, 16.2%) compared to those who were privileged with these benefits. Regarding training, (n = 304, 67%) of the participating healthcare providers were trained on the risk and hazards of COVID-19 and were more (n = 41, 13.5) affected by insomnia. Most health professionals were provided with PPE, but (n = 148 32.6%) were not satisfied with the quality and developed significant insomnia (n = 26, 17.6%). Among these factors lacking service benefits, diminished working hours, not getting sick leave, no risk allowance and accommodation facility, and dissatisfaction with PPE quality were significantly associated with the development of Insomnia among HCWs. Whereas type of employment, whether fixed or short-term contractual, written appointment letter, overtime night duty, arrangement of nutritious food, and training on risk and hazards of COVID-19 were not among the statistically significant predictors of insomnia.

**Table 2:** Association between insomnia and job stressors

Question on Job Stressors	Total Number of Observations N	Insomnia n (%)	p-value
*Types of employment*			
Permanent	248 (54.6)	35 (14.1)	0.349
Short-term Contractual (< 1 year)	206 (45.4)	23 (11.2)	
*Were you provided with written appointment letter*			
Yes	429 (94.5)	56 (13.1)	0.462
No	25 (5.5)	2 (8.0)	
*Is there any service benefit*			
Yes	330 (72.7)	32 (9.7)	0.001**
No	124 (27.3)	26 (21.0)	
*Provision of overtime*			
Yes	214 (47.1)	29 (13.6)	0.640
No	240 (52.9)	29 (12.1)	
*Changes in working hour due to COVID 19*			
Increased	365 (80.4)	49 (13.4)	0.036**
Decreased	8 (1.8)	3 (37.5)	
Unchanged	81 (17.8)	6 (7.4)	
*Sick leave*			
Yes	304 (67.0)	20 (6.6)	< 0.001***
No	150 (33.0)	38 (25.3)	
*Received any COVID 19 Risk allowance*			
Available	146 (32.2)	8 (5.5)	0.001**
Not available	308 (67.8)	50 (16.2)	
*Special nutritious food arrangement by employer*			
Yes	195 (43.0)	21 (10.8)	0.267
No	259 (57.0)	37 (14.3)	
*Accommodation provided by employer*			
Yes	261 (57.5)	24 (9.2)	0.008**
No	193 (42.5)	34 (17.6)	
*Quality and satisfaction regarding PPEs*			
Satisfied with the quality	306 (67.4)	32 (10.5)	0.033**
Not satisfied with the quality	48 (32.6)	26 (17.6)	
*Any training provided on risks and hazards*			
Yes	304 (67.0)	41 (13.5)	0.518
No	150 (33.0)	17 (11.3)	

***=p < 0.001; **=p < 0.05

Table 3 contains the results of binary regression analysis, which revealed the independent risk factors of Insomnia in HCWs. Participants diagnosed previously with COVID-19 were about 2.6 times (OR = 2.596, 95% CI = 1.248–5.399) more prone to develop insomnia than those who remained COVID-19-negative, pointing at negative experience influencing insomnia. Participants entitled to sick leave showed nearly four times (OR = 0.248, 95% CI = 0.116, 0.532) lower odds than not having sick leaves. With almost 2.72 times lower odds (OR = 0.367, 95% CI = 0.124.1.081), not getting any risk allowance, and with 1.9 times higher odds, provision of training on risk and hazard were the factors that analytically had a significant association with causing insomnia.

**Table 3:** Associated factors identified by multiple logistic regression analysis

Factors	Adjusted for other covariates	
	OR	95% CI
*Age of the participants*	0.977	0.909–1.050
*Gender of the participants*		
Male	0.980	0.435–2.208
Female	1 [Reference]	
*Designation*		
Doctor	0.964	0.217–4.272
Nurse	2.002	0.621–6.450
Others	1 [Reference]	
*Highest educational level*		
Undergraduate	0.499	0.187–1.331
Graduate and above	1 [Reference]	
*Marital status*		
Married	0.741	0.378–1.453
Unmarried	1 [Reference]	
*Total working experience*	1.008	0.896–1.134
*Have you ever received any training regarding COVID 19*		
Yes	0.665	0.284–1.559
No	1 [Reference]	
*Have you ever been diagnosed with COVID 19 disease*		
Yes	2.596	1.248–5.399**
No	1 [Reference]	
*Smoking history*		
Yes	1.192	0.465–3.053
No	1 [Reference]	
*Types of employment*		
Permanent	1.175	0.581–2.372
Short-term Contractual (< 1 year)	1 [Reference]	
*Availability of service benefit*		
Yes	0.533	0.249–1.143
No	1 [Reference]	
*Provision of overtime*		
Yes	0.608	0.303–1.219
No	1 [Reference]	
*Sick Leave*		
Yes	0.248	0.116–0.532***
No	1 [Reference]	
*Received any COVID 19 Risk allowance*		
Yes	0.367	0.124–1.081
No	1 [Reference]	
*Special nutritious food arrangement by employer*		
Yes	1.965	0.760–5.076
No	1 [Reference]	
*Accommodation provided by employer*		
Yes	0.510	0.219–1.192
No	1 [Reference]	
*Any training provided on risks and hazards*		
Yes	1.923	0.934–3.958
No	1 [Reference]	

***=p < 0.001; **=p < 0.05

## Discussion

The unanticipated pandemic moulded the physical and psychological status of millions of people across the planet. Being the vulnerable group, frontline HCWs experienced the most distressing episodes of the COVID-19 outbreak. The continuous exposure to morbid patients, fear of contagion, and overburdened death toll propelled the HCWs towards many negative emotions. The culmination of such negativity results in sleep disturbances and insomnia. Several studies have discovered that HCWs worldwide generally suffer more from sleep disturbances than any other occupation group. Disrupted circadian rhythm, fear of being infected and infecting family members are some reasons found in the literature. In a pandemic, exacerbations of these risk factors occur in ten folds affecting the HCWs, the front liners [6, 20, 21].

Bangladesh is a low and middle-income country with a disadvantaged health sector. The prevalence of insomnia and its associated factors was explored in the frontline fighters working in highly infectious COVID care units. Among the 454 HCWs, doctors and nurses were preeminent, comprising 44.9% and 33.9% of overall respondents, respectively.61.5% of the attendees were females, 68.3% completed their higher studies, and three-fourths (75.3%) were Muslims. A cross-sectional study was conducted using the Insomnia severity index and a higher prevalence of clinically significant insomnia was reported by 12.78% HCWs (n = 58). A similar prevalence of 18.2% was found in a study in China [20].

Doctors and nurses were the worst sufferings from insomnia, 16.2% and 13.6%, respectively, among health care workers. Sahin et al. conducted an identic study in Turkey and found that HCWs in regular direct contact with COVID patients are prone to insomnia compared to second-liners [11]. A higher prevalence was observed among the graduates (13.9%) in our study, indicating the raised risk perception level. A study conducted in China found the opposite findings, where the less educated were more at risk due to fear of the unknown and uncertainty [22]. This study found male participants more affected than females (14.3% vs. 11.8%) with significant insomnia. However, some studies found no differences, whereas others found the female gender a risk factor for insomnia and other psychological issues [20, 21, 23].

Interestingly, although followers of Islam were the majority in our study, they were less distressed with sleep problems (12.3%). The married HCWs were also less affected (10.9%) by sleep disturbance, probably due to their partner’s mental and physical support. Similar results can be observed in previous studies where single HCWs were much more affected by sleep problems [11, 24].

More significant insomnia was indicated in this study among doctors than nurses; this could be due to their higher educational status, risk perception, and overall ascended knowledge about the current situation than other groups. A meta-analysis of seven cross-sectional studies revealed a higher prevalence of Insomnia in doctors (41.6%) than in nurses (34.8%) [25]. Two separate Chinese studies have also found that being a doctor is related to developing Insomnia [20, 21]. In contrast, Jianbo et al. [5] and Herrero San Martin et al. [22] found in their respective studies that being a nurse is a risk factor for developing insomnia as nurses spend the most time alongside COVID patients.

HCWs who attended COVID-19-related training programs (13.3% of the respondents) were more likely to suffer from insomnia. Supporting claims can be found in a survey conducted by Zhang et al. on insomnia and related social psychological factors of healthcare workers [26]. Additionally, medical staff who were previously diagnosed as COVID-19-positive and had the chronic disease were at higher risk of developing insomnia in that study. Likewise, in the present study, two variables were linked to a higher prevalence of insomnia: ever been diagnosed with COVID-19 (16.7%) and having a chronic disease (15.6%). After adjusting for other covariates, binary logistic analysis portrayed 2.5-fold higher odds (OR = 2.596, 95% CI = 1.248, 5.399) of developing insomnia among previously confirmed COVID-19-positive cases, possibly due to their bitter experience, stress and fear of the catastrophic consequences of COVID-19 and the subsequent complications of a second infection. This study found a 14.9% higher prevalence of insomnia among 67 smokers than among non-smokers. Lee et al. [23] found a significant association between increased smoking and drinking with insomnia while investigating the relationship between mental health problems and unhealthy behaviour among medical staff.

Binary logistic regression found a lack of risk allowance and sick leave to be significantly associated with insomnia. Moreover, lack of proper rest with less time to attain the proper mental stability owing to increased workload combined with lack of sick leave puts significant physical strain, as found in this study. Supporting claims have been found that lack of support and negative behaviour is significantly associated with Insomnia development [26]. Zou et al. concluded in their study that organizational support could have a protective effect regarding insomnia and vice versa [16]. Logistics also highlighted that HCWs lacking training on risk and hazards are significantly more likely to suffer from sleep problems. Other studies have found evidence that positive reinforcements like training can work against mental health problems such as insomnia [26, 27].

Compared to other similar studies, we conducted face-to-face interviews, maintaining proper protective etiquette. The limitations of this study are the lack of causal inferences, as ours is a cross-sectional study, and the lack of generalizability due to movement restrictions during COVID may affect the final result. A longitudinal study with a more generalized sample is needed to resolve the gaps.

## Conclusion

In this endeavour, we have identified that ever being diagnosed with COVID-19 in the past and job-related stressors such as scarcity of sick leave, pandemic-specific risk allowance, or insurance for the family benefit and prior training on risk and hazard of this catastrophe are top influencers for developing insomnia. This study also suggests that job-related inconveniences and unknown consequences during a new pandemic affect the frontline fighters’ mental health and sleep pattern. Healing the healers should be the prime concern now, which could achieve by establishing a resilient work environment. The immediate impact of the pandemic suggests the urgency to generate and implement collaborative interventions to help HCWs cope with the repercussions of this prodigious crisis. Concerned authorities should be more mindful of the current condition of the valuable defence against COVID-19. Specific interventional research is essential immediately to mitigate the mental health impacts on healthcare workers and help them cope with this ruination.

## Data Availability

The data underlying the results presented in this study will be provided on reasonable request to Dr. Delwer H. Hawlader. Email: mohammad.hawlader@northsouth.edu.
